# Predictors of Posterior Reversible Encephalopathy Syndrome (PRES) in Women With Pre-eclampsia/Eclampsia: A Retrospective Analysis

**DOI:** 10.7759/cureus.31459

**Published:** 2022-11-13

**Authors:** Anupama Bahadur, Rajlaxmi Mundhra, Rajni Singh, Juhi Mishra, Gayatri Suresh, Shweta Jaiswal, Dibna Sinha, Mritunjai Singh

**Affiliations:** 1 Obstetrics and Gynaecology, All India Institute of Medical Sciences, Rishikesh, Rishikesh, IND; 2 Neurology, All India Institute of Medical Sciences, Rishikesh, Rishikesh, IND

**Keywords:** neuro radiology, posterior reversible encephalopathy syndrome (pres), hypertension in pregnancy, preeclampsia-eclampsia, pre-eclampsia

## Abstract

Context: Posterior reversible encephalopathy syndrome (PRES) is a clinical-radiological entity characterized by acute neurological symptoms with reversible subcortical vasogenic brain edema. One of the most common risk factors is pre-eclampsia/eclampsia.

Aims: This study aimed to compare the clinical and radiological characteristics of PRES with those without PRES in patients with pre-eclampsia/eclampsia and attempts to find independent predictors of PRES.

Methods and Materials: This was a single-center, retrospective study. Fifty-three female patients admitted to the Department of Obstetrics & Gynaecology, AIIMS Rishikesh between 2018 and 2021 with severe pre-eclampsia/eclampsia were included. Brain imaging was done to confirm the diagnosis of PRES. Baseline characteristics between patients whose imaging was suggestive and not suggestive of PRES were compared.

Results: Fifty-three patients with pre-eclampsia/eclampsia were included in the analysis. The median age and period of gestation of the study population were 28 (range 19-37) years and 36.6 (range 24.2-41.5) weeks respectively. Twelve patients (22.6%) had eclampsia, and 41 (87.4%) had pre-eclampsia of which 28 (52.8%) had severe pre-eclampsia. Twelve patients were diagnosed with PRES. Patients with PRES were significantly younger with a median age of 23 [range 20-30 vs 29 (range 19-37; p = 0.005) years], and more likely to be primiparous (91.7% vs 36.6%; p < 0.001) compared to those without PRES. PRES was significantly more common in patients with eclampsia. Of 12 patients with eclampsia, nine (75%) had evidence of PRES. The maternal and fetal outcome, however, was similar in both groups. Patients with PRES were more likely to have poor sensorium compared to those without PRES (83.3% vs 5.3%; p < 0.01). Eclampsia was found in the independent predictor of PRES (odds ratio, OR 20.9; 95% confidence interval, CI 3.0-147.0, p = 0.02).

Conclusions: In this study, patients with PRES were younger and significantly more likely to be primiparous and have eclampsia compared to those without PRES. Headache followed by seizures and altered sensorium were the most common clinical manifestations and subcortical white matter hyperintensities involving fronto-parieto-occipital lobes were the most common radiological finding. Eclampsia emerged as an independent risk factor for PRES.

## Introduction

Posterior reversible encephalopathy syndrome (PRES) is a clinical-radiological entity characterized by acute neurological symptoms (e.g., seizures, encephalopathy, headache, and visual disturbances) with reversible subcortical vasogenic brain edema. It was first described by Hinchey et al. (1996) in a case series [1]. The most common risk factors are renal failure, blood pressure fluctuations, cytotoxic drugs, autoimmune disorders, and pre-eclampsia/eclampsia.

Pre-eclampsia is described as new-onset hypertension after 20 weeks of gestation, associated with proteinuria (>300 mg/dL in 24 h) or with signs of end-organ dysfunction [2]. Eclampsia is characterized by new onset seizures/convulsions in a woman with pre-eclampsia in the absence of any other cause [3]. Both pre-eclampsia and eclampsia may be associated with PRES [4-6]. Recent studies have shown conflicting results on the association of eclampsia with PRES. A retrospective cohort study of all patients with eclampsia between 2001 and 2010 showed that 97.9% (46/47 patients) patients had features of PRES on brain imaging [7], which is further confirmed by other studies [8-9], demonstrating that PRES is a core component of the pathogenesis of eclampsia. On the contrary, other studies showed that only half of the imaged patients with eclampsia had PRES [4, 6, 10-11]. Most of the previous studies are retrospective in nature with the risk of selection bias. There is a paucity of literature on the comparison of clinical and radiological characteristics among patients with PRES to those without PRES in a prospective cohort study [4, 11]. The present study intends to compare the clinical and radiological characteristics of PRES with those without PRES in patients with pre-eclampsia/eclampsia and attempts to find independent predictors of PRES. This study might help in the early recognition of pre-eclamptic/eclamptic women at risk of developing PRES.

## Materials and methods

This single-center observational study was conducted in unit two of the obstetrics and gynecology department during 2018-2021. 

*Inclusion criteria*: Consecutive women diagnosed with pre-eclampsia/eclampsia in the antepartum or postpartum period who were admitted to the department of obstetrics and gynecology during 2018-2021 were included. The diagnosis of pre-eclampsia was based upon criteria set down by the American College of Obstetricians & Gynecologists [3] as mentioned in Table 1. The occurrence of manifest seizures confirmed the diagnosis of eclampsia in these patients.

**Table 1:** Criteria for diagnosis of pre-eclampsia (ACOG 2020) [3].

Systolic blood pressure ≥140 mmHg or diastolic blood pressure ≥90 mmHg on at least two occasions at least 4 h apart after 20 weeks of gestation in a previously normotensive patient AND the new onset of one or more of the following:
Proteinuria ≥0.3 g in a 24-h urine specimen or protein/creatinine ratio ≥0.3 (mg/mg) (30 mg/mmol) in a random urine specimen or dipstick ≥2+ if a quantitative measurement is unavailable
Platelet count <100,000/microL
Serum creatinine >1.1 mg/dL (97.2 micromol/L) or doubling of the creatinine concentration in the absence of other renal diseases
Liver transaminases at least twice the upper limit of the normal concentrations for the local laboratory
Pulmonary edema
New-onset and persistent headache
Visual symptoms (eg, blurred vision, flashing lights or sparks, scotomata)

*Exclusion criteria*: We excluded patients with a history of neurologic disorders (e.g., epilepsy, cerebrovascular accident, cerebral hemorrhage, demyelinating disorders, intracranial infections, cranial surgery). We also excluded patients developing pre-eclampsia/eclampsia after being discharged, but within the puerperal period for logistic reasons.

Clinical examination and investigations

Detailed obstetrical history including the date of the last menstrual cycle (to calculate the period of gestation), parity, and history of previous cesarean surgery was taken. The period of gestation was also confirmed by ultrasonography. Every patient underwent a detailed clinical examination. Blood pressure was measured every four hours in all the patients, as a part of institute protocol, and every hour in those with elevated blood pressure readings. Symptoms suggestive of PRES such as headache, visual blurring, and seizures were noted. Patients were examined for focal neurological deficits and the sensorium was assessed using Glasgow Coma Scale (GCS). 

Imaging and diagnosis of PRES

Brain imaging was done to exclude alternative diagnoses, and also to confirm the diagnosis of PRES. Cranial MRI and MR angiography (MRA) and venography were carried out using a 3T scanner (Signa GE Medical System, Wisconsin, USA). The decision to order MRI in a patient with pre-eclampsia/eclampsia was at the discretion of the attending physician. Only symptomatic patients underwent MRI with MRA and venography, as it was considered unethical for an asymptomatic patient to undergo MRI with inherent risk to the fetus and mother [MOU1] [MOU2] [12]. Contrast gadodiamide 0.1 nmol/kg intravenously was used wherever indicated [MOU3]. MRI brain included the following sequences: T1W, T2W, fluid attenuated inversion recovery (FLAIR), and diffusion weighted imaging (DWI) with corresponding apparent diffusion coefficient (ADC) values. Susceptibility weighted imaging (SWI) sequences were obtained to rule out hemorrhage. 

The diagnosis of PRES was based on the presence of vasogenic edema, characterized by T1 iso/hypo and T2W/FLAIR hyper-intensity and the absence of secondary causes. The findings were confirmed by two independent radiologists blinded for the clinical details. The diagnosis of infarction was based on MRI showing iso-intense to hypointense on T1W and hyperintense on T2W/ FLAIR, and/or restriction on DWI.

Management 

There are no randomized controlled trials on the interventions for treating PRES, and the mainstay of management is supportive care, hydration, dyselectrolytemia correction along with control of hypertension [13]. Removal of the inciting factor is most important, and it is important to expedite the delivery of the fetus and placenta in the pregnant patient. The route of delivery depended on the clinical situation. Magnesium sulfate remains the drug of choice for controlling seizures in pregnancy. Antihypertensive measures should focus on reducing blood pressure by 20%-25% with a target mean arterial pressure (MAP) of 105-125 mmHg. Most patients required intensive care unit (ICU) care in view of prolonged ventilatory care, continuous antihypertensive infusions, and intensive monitoring. 

Statistical method 

Continuous and normally distributed variables were represented as mean ± SD (standard deviation) while continuous but skewed variables were represented as median and range. Statistical significance was defined as a two-tailed p-value < 0.05. For normally distributed continuous variables, an independent t-test and for skewed variables, Mann Whitney U test was used. Chi-square or Fisher’s exact test was used to compare the categorical variables. Statistical analyses were performed using SPSS version 20.0 software (SPSS Inc., Chicago, IL, USA).

## Results

Fifty-eight patients with pre-eclampsia/eclampsia were included in the study. Three patients had cortical venous thrombosis, one patient each had intracranial space-occupying lesions and neuro-cysticercosis, hence were excluded from the study. The final analysis was, therefore, based on 53 patients. The median age of the cohort was 28 (range 19-37) years with a median period of gestation 36.6 (range 24.2-41.5) weeks. Twelve patients (22.6%) had eclampsia, and 41 (87.4%) had pre-eclampsia of which 28 (52.8%) had severe pre-eclampsia. Twelve patients were diagnosed with PRES. 

Comparison of clinical characteristics between patients with and without PRES

Patients with PRES were significantly younger with a median age of 23 [range 20-30 vs 29 (range 19-37; p = 0.005)] years, and more likely to be primiparous (91.7% vs 36.6%; p < 0.001) compared to those without PRES. PRES was significantly more common in patients with eclampsia. Of 12 patients with eclampsia, 9 (75%) had evidence of PRES. The maternal and fetal outcome, however, was similar in both groups. The details of baseline characteristics have been shown in Table 2.

**Table 2:** Comparison of baseline characteristics of patients with pre-eclampsia/eclampsia with and without PRES. PRES, posterior reversible encephalopathy syndrome; SBP, systolic blood pressure; DBP, diastolic blood pressure; ALT, alanine transaminase; AST, aspartate aminotransferase; ALP, alkaline phosphatase

Characteristics	PRES N = 12	No-PRES N = 41	p
Age (years)	23 (20–30)	29 (19–37)	0.005
Primipara	11 (91.7)	15 (36.6)	<0.01
Period of gestation (weeks)	36.1 (28.3–39.0)	37.1 (24.2–41.5)	0.17
SBP (mmHg)	160 (130–190)	160 (110–194)	0.76
DBP (mmHg)	100 (80–130)	100 (60–130)	0.93
Eclampsia severe pre-eclampsia mild pre-eclampsia	9 (75.0) 3 (25.0) 0 (0.0)	3 (7.3) 25 (61.0) 13 (31.7)	<0.01
Admission to delivery (days)	1 (1–6)	1 (1–9)	0.86
Mode of delivery (cesarean section)	11 (100)	37 (90.2)	0.56
Fetal deaths	3 (25.0)	6 (14.6)	0.41
Epigastric pain	4 (33.3)	2 (4.9)	0.02
Pedal edema	8 (66.7)	3 (7.3)	<0.01
Oliguria	3 (25.0)	1 (2.4)	0.03
Renal dysfunction	1 (8.3)	1 (2.4)	0.41
Thrombocytopenia	1 (8.3)	1 (2.4)	0.41
ALT (U/L)	253 (60–325)	32 (12–436)	0.05
AST(U/L)	142 (40–200)	40 (16–488)	0.10
ALP (IU/L)	506 (390–622)	285 (89–1437)	0.44
Bilirubin (total) (mg/dL)	1.2 (0.7–1.7)	0.7 (0.68–1.8)	0.09

Thirty-one out of 53 patients having one or more neurological symptoms underwent an MRI brain. The indications for imaging were headache in 25/53 (47.2%), blurring of vision in 11/53 (20.8%), altered sensorium in 11/53 (20.8%), seizures in 12/53 (22.6%), and focal neurological deficits in 2 (3.8%) patients. Most patients had generalized tonic-clonic seizures, which had right focal with secondary generalized seizures. None had status epilepticus, however.

**Figure 1:**
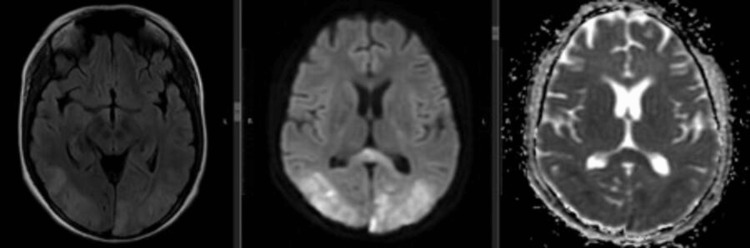
MRI brain axial section showing FLAIR (B) hyper-intensities in the parieto-occipital region, with diffusion restriction (B & C) suggestive of cytotoxic edema (infarct), intermixed with vasogenic edema in a patient with eclampsia with symptoms of cortical blindness only. MRI was suggestive of PRES with infarct. FLAIR, fluid-attenuated inversion recovery; PRES, posterior reversible encephalopathy syndrome

Patients with PRES were more likely to have poor sensorium compared to those without PRES (83.3% vs 5.3%; p < 0.01). Two patients with PRES also had focal neurological deficits in the form of hemiparesis. Other symptoms such as headache and visual blurring were similar in those with PRES to those without. One of our patients had only cortical blindness (Figure 1). Details have been shown in Table 3. 

**Table 3:** Comparison of clinical and radiological findings of patients with pre-eclampsia/eclampsia with and without PRES. PRES, posterior reversible encephalopathy syndrome

Characteristics	PRES N = 12	No-PRES N = 19	p
Indications for MRI brain			
Headache	8 (66.7)	17 (89.5)	0.17
Visual symptoms	5 (41.7)	6 (31.6)	0.71
Altered sensorium	10 (83.3)	1 (5.3)	<0.01
Focal deficits	2 (15.4)	0 (0.0)	-
Seizures	9 (75.0)	3 (15.8)	0.002
Radiological findings			
Gray matter	6 (50.0)	2 (10.5)	0.03
Basal ganglia involvement	2 (16.7)	0 (0)	-
Infarct	5 (41.7)	1 (5.3)	0.02
White matter	9 (75)	1 (5.3)	<0.01
Frontal hyperintensities	5 (41.7)	1 (5.3)	0.02
Parietal hyperintensities	6 (50.0)	0 (0.0)	-
Occipital hyperintensities	4 (33.3)	0 (0.0)	-
Temporal hyperintensities	1 (8.3)	0 (0.0)	-
Hemorrhage	1 (8.3)	0 (0.0)	-

Imaging characteristics

**Figure 2:**
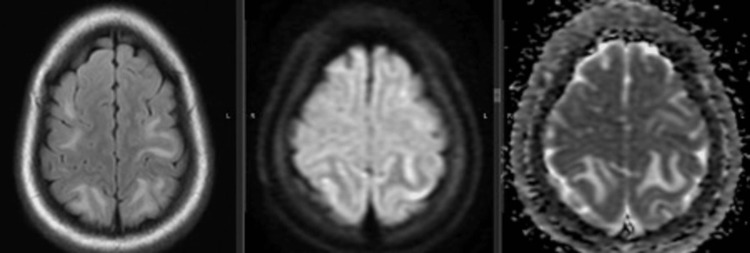
MRI brain axial section showing FLAIR (B) hyper-intensities in the parieto-occipital region extending to frontal lobes, without corresponding diffusion restriction (B & C), suggestive of vasogenic edema in a patient with eclampsia. MRI was suggestive of PRES. FLAIR, fluid-attenuated inversion recovery; PRES, posterior reversible encephalopathy syndrome

Overall, the most common abnormality noted on brain imaging was subcortical white matter hyper-intensities without diffusion restriction suggestive of vasogenic edema. Subcortical white matter involvement was seen in 15 of 31 patients undergoing brain imaging, significantly more common in patients with PRES compared to those without (91.7% vs 21.1%; p = 0.001). The most common location for sub-cortical hyper-intensities was frontal followed by parietal and occipital. While frontal lobe lesions were also present in those without PRES, parieto-occipital lesions were seen exclusively in those with PRES (Figure 2). Infarctions and gray matter involvement were also significantly more common in patients with PRES (Figure 1). The comparison of detailed radiological findings is shown in Table 3.

Predictors of PRES

Multivariate logistic regression analysis using variables such as age, parity, and presence or absence of eclampsia (all variables with p < 0.1 on the univariate analysis) was done to find the independent predictors of PRES. The presence of pedal edema and epigastric distress was excluded from the regression model to avoid multi-collinearity, as they are likely to be associated with severe pre-eclampsia/eclampsia. On regression analysis, eclampsia was found to be the independent predictor of PRES (OR 20.9; 95% CI 3.0-147.0, p = 0.02). Age (OR 1.3; 95% CI 0.95-1.67, p = 0.12) and parity (OR 6.8; 95% CI 0.5-91.3, p = 0.15) did not predict PRES. 

## Discussion

In this study, patients with PRES were younger and significantly more likely to be primiparous and have eclampsia compared to those without PRES. Headache followed by seizures and altered sensorium were the most common clinical manifestations and subcortical white matter hyperintensities involving fronto-parieto-occiptal lobes were the most common radiological finding. Eclampsia emerged as an independent risk factor for PRES. 

Various retrospective studies have found that almost 92%-100% of eclamptic women had PRES [7, 9, 14]. It has also been suggested that PRES is an integral component of eclampsia [7]. However, the same could not be recapitulated in other studies which showed that only 41%-63% of imaged eclamptic women had clinical and radiologic findings of PRES [6, 10]. Most of these studies were retrospective in nature, limited partly by selection bias and/or inconsistent diagnostic workup. Few prospective studies have been done in this regard. In a prospective observational study of 104 women with eclampsia undergoing MRI, Verma et al. [4], found PRES in 71.4% of patients with eclampsia. The findings of our study are in concordance with the later two studies. We noted PRES in 9/12 (75%) patients with eclampsia. The occurrence of PRES in 3/28 (10.7%) of patients with severe pre-eclampsia in our study underscores the fact that PRES can occur not only in eclamptic women but also in those with severe pre-eclampsia emphasizing the importance of having a high index of suspicion among pre-eclamptic women with neurologic signs [9, 11, 15]. Similar findings have been noted in previous studies [4, 6, 11]. This raises an important issue of performing routine MRI brain in those with pre-eclampsia. We, however, do not recommend routine neuroimaging in all patients with pre-eclampsia. Rather, neuro-imaging should be limited to preeclamptic patients with neurologic symptoms, because neither MRI diagnosis of PRES changed the final management nor did the diagnosis of PRES affect the fetal or maternal outcome in our study.

We found that PRES was more common in younger age and in nulliparous women similar to those reported in previous studies [4, 6, 11]. However, the absolute systolic and diastolic blood pressures (BPs) were not different in patients with or without PRES. It could be due to the fact that most of our patients were already on anti-hypertensive treatment at the time of admission. Moreover, a lack of association between blood pressure and PRES has also been reported previously in multiple studies [4-5, 7, 10, 16]. In fact, normal or even lower BPs have been found to be associated with PRES [17]. It can thus be suggested that aggressive blood pressure management should be the mainstay of treatment in women with hypertension in pregnancy as recommended by the International Society for the Study of Hypertension in Pregnancy (ISSHP) and American College of Obstetricians and Gynecologists (2013) [2, 18]. The most common symptoms of PRES in our study were altered sensorium (83.3%) followed by seizures (75.0%) and headache (66.7%). Similar proportions have been reported in multiple other studies [4, 6-7]. Visual symptoms, though considered typical of PRES were noted in only 41.7% of our patients. The inability to assess visual symptoms in unconscious patients could be one of the reasons for a lower proportion of visual symptoms in our cohort. Radiologically, we concur with the previous studies that parieto-occipital sub-cortical white matter hyper-intensities secondary to vasogenic edema were specific findings on MRI brain in patients with PRES [4, 19]. In our study, infarcts were noted in 41.7% of patients, and basal ganglia involvement in 16.7% of patients similar to those reported previously [4]. However, the proportion of patients with radiological findings in our study was less common than those reported by Fisher et al. (2016) and Liman et al. (2012) [6, 20]. 

Posterior reversible encephalopathy syndrome is characterized by endothelial injury secondary to abrupt blood pressure changes. Endothelial injury results in cytokine cascade and breakdown of the blood-brain barrier and subsequent brain edema. PRES is generally reversible, both radiographically and clinically, and generally has a favorable prognosis as evidenced by our study. although we did not look for the reversibility of the radiological findings; however, there were no maternal deaths and fetal outcome was comparable between the groups. Various studies have shown vision impairment, younger age, primigravida, unbooked status, and the presence of eclampsia [4-6, 16]. We, however, found only eclampsia as an independent predictor for PRES. This study underscores the fact that patients with eclampsia must be followed closely.

Limitations

This study is limited by a small sample size. However, it is difficult to recruit a large number of patients from a single center. Thus, large multi-center studies are needed to confirm our results. Also, this study has been done at a tertiary care referral hospital, limiting its generalizability. Further, we did not perform MRI on all the patients and limited ourselves to only those with symptoms due to ethical reasons. This could have undermined the probability of finding radiological abnormalities in those without symptoms. 

## Conclusions

In this study, patients with PRES were younger and significantly more likely to be primiparous and have eclampsia compared to those without PRES. Headache followed by seizures and altered sensorium were the most common clinical manifestations and subcortical white matter hyperintensities involving fronto-parieto-occipital lobes were the most common radiological finding. Eclampsia emerged as an independent risk factor for PRES.
